# Ampa Receptor Subunit Expression in the Endoplasmic Reticulum in Frontal Cortex of Elderly Patients with Schizophrenia

**DOI:** 10.1371/journal.pone.0039190

**Published:** 2012-06-14

**Authors:** John C. Hammond, James H. Meador-Woodruff, Vahram Haroutunian, Robert E. McCullumsmith

**Affiliations:** 1 Department of Neurobiology, University of Alabama Birmingham, Birmingham, Alabama, United States of America; 2 Department of Psychiatry and Behavioral Neurobiology, University of Alabama Birmingham, Birmingham, Alabama, United States of America; 3 Department of Psychiatry, Mount Sinai School of Medicine, New York, New York, United States of America; University of Queensland, Australia

## Abstract

Several lines of evidence indicate altered trafficking of α-amino-3-hydroxyl-5-methyl-4-isoxazole-propionate (AMPA) receptors in schizophrenia. Previous reports have shown potential changes in the trafficking of AMPA receptors based on subunit expression of endosomes, subcellular organelles located near post-synaptic sites. We hypothesized that alterations in AMPA receptor trafficking through the endoplasmic reticulum (ER) may also be altered in schizophrenia. Accordingly, we developed a technique to isolate and measure content of the ER from postmortem brain tissue. We used Western blot and electron microscopy to show that we isolated an ER enriched fraction. We found no changes in the expression of the AMPA receptor subunits, GluR1–4, in the ER from the dorsolateral prefrontal cortex in schizophrenia. These data suggest that AMPA receptor trafficking through the ER is largely intact in schizophrenia.

## Introduction

Schizophrenia, a severe mental illness, may be linked to abnormalities of glutamate receptor neurotransmission and expression [1]–[3]. Recent findings support the hypothesis that alterations in glutamate neurotransmission may be part of the underlying pathophysiology of schizophrenia [4]. Recent evidence also implicates abnormalities in trafficking of AMPA-type glutamate receptors in this illness [5]–[7]. Alterations of AMPA receptor interacting proteins, as well as altered subcellular localization of AMPA receptor subunits, have also been described [5], [6].

The AMPA receptor consists of four subunits, GluR1–4, that are typically assembled as dimers into a tetrameric complex in the endoplasmic reticulum (ER) [8], [9]. AMPA receptor subunit composition influences the rate of transit of AMPA receptors through the ER, with AMPA receptors containing GluR1/GluR2 subunits trafficking faster than GluR2/GluR3 containing receptors [10]. Additionally, AMPA receptor interacting proteins alter the rate of trafficking of AMPA receptors through the ER and to the synapse. Synapse-associated protein 97 (SAP97) binds to the PDZ (postsynaptic density 95/Discs large/zona occludens-1) domain of GluR1 in GluR1/GluR2 hetero-oligomers for fast trafficking from the ER to the Golgi [11], [12]. Protein interacting with C Kinase 1 (PICK1) binds to the C-terminal domain of GluR2 in GluR2/GluR3 containing receptors, leading to slower exit from the ER [10]. Trafficking of assembled AMPA receptors from the ER to the distal dendrites occurs along the cytoskeletal spine. Glutamate Receptor Interacting Protein 1 (GRIP1) interacts with GluR2 containing receptors and kinesin motor proteins to bring assembled AMPA receptors along the dendrites to the synapse [13]–[15]. AMPA receptors in the distal dendrites can be surface expressed at the synapse in an activity-dependent manner [16], [17]. Surface expressed AMPA receptors can be removed from and returned to the synapse in a tightly regulated endosomal system, facilitating changes in synaptic strength [18].

Previous studies have measured the expression of AMPA receptor protein and mRNA at a regional level and found no changes in schizophrenia [19]–[21]. Recent studies have examined expression of AMPA interacting proteins and subcellular localization of AMPA receptors in schizophrenia [5], [7], [22]. In postmortem human dorsolateral prefrontal cortex samples, we have previously found an increase in the AMPA receptor GluR1 subunit in early endosomes and no difference in the expression of AMPA receptor subunits in late endosomes [5], [22]. Additionally, SAP97 and GRIP1 were increased in schizophrenia, suggesting an increase in the rate of forward trafficking of AMPA receptors from the ER to the synapse [5].

Based on these findings, we hypothesized that accelerated proximal forward trafficking and decreased ER retention of AMPA receptor subunits may be associated with the pathophysiology of schizophrenia. To further test this hypothesis, we developed a technique to isolate ER from postmortem human brain tissue, characterized this ER fraction, and measured the expression of AMPA receptor subunits in this fraction from subjects with schizophrenia and a comparison group.

## Results

### Endoplasmic Reticulum Isolation and Characterization

**Figure 1:**
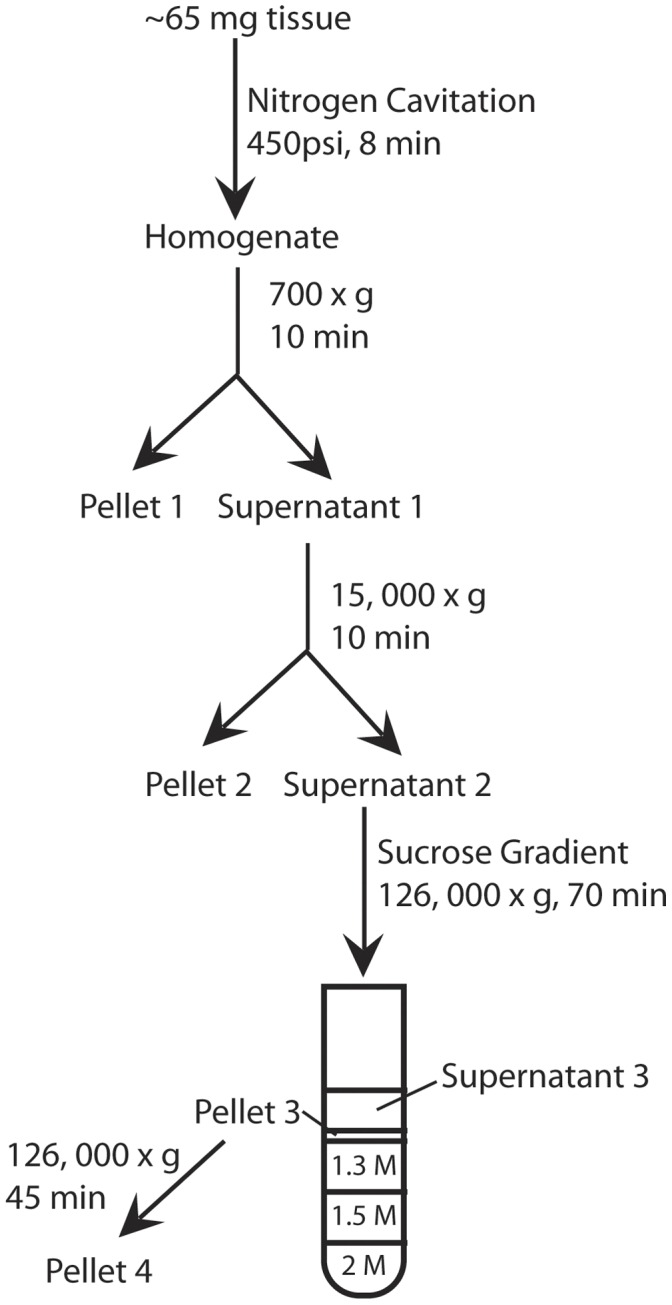
Flow chart of endoplasmic reticulum isolation. Nitrogen cavitation of tissue was followed by a series of centrifugation steps and sucrose density gradient centrifugation.

**Figure 2:**
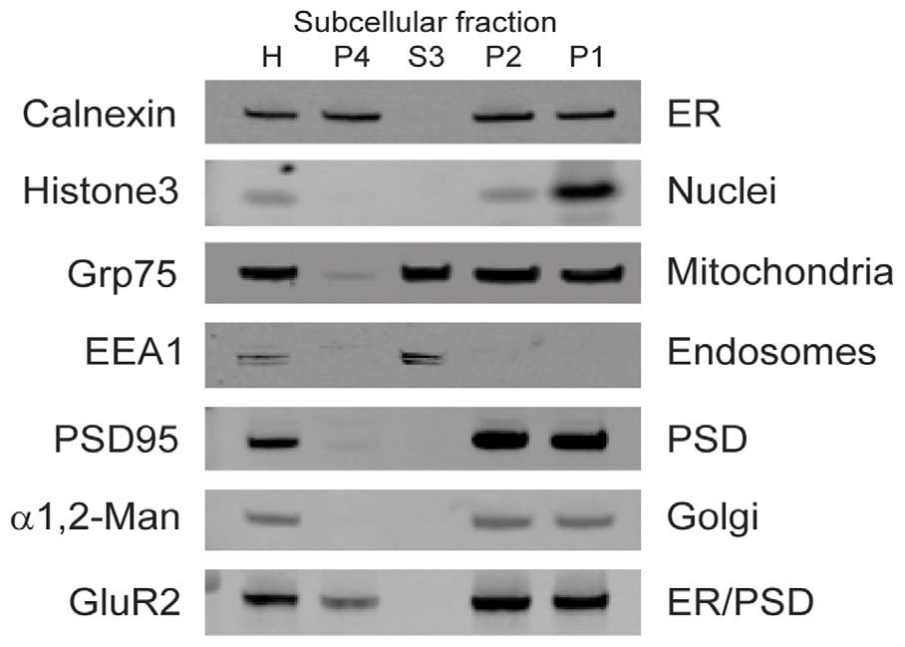
Neurochemical characterization of subcellular fractions isolated from postmortem human frontal cortex. Fractions enriched for endoplasmic reticulum (P4), cytosol and ‘light’ membranes (S3), mitochondria (P2), and nuclei (P1) were assayed by Western blot for compartment-specific markers: Calnexin (ER), histone3 (Nuc), glucose regulated 75 kDa protein (Grp75, Mito), early endosome antigen 1 (EEA1, endosomes), postsynaptic density 95 (PSD95, PSD), and α1,2 mannosidase (α1,2-Man, Golgi). The PSD segregated with the other ‘heavy’ membranes in the Mit and Nuc fractions. As expected, AMPA receptor subunit 2 (GluR2) was detected in the ER and fractions containing the PSD.

We used differential sucrose gradient centrifugation to obtain an ER enriched fraction from postmortem brain tissue (Figure 1). In addition to an enriched ER fraction, we obtained 3 other fractions. To verify and characterize the identity of each fraction, we used a wide screen of antibodies specific for various subcellular compartments (Figure 2). As expected, bands for each antibody were present in total brain homogenate. In the nuclear fraction (pellet 1), we detected calnexin (an ER marker), histone3 (a nuclear marker), Grp75 (a mitochondrial marker), PSD95 (a post-synaptic density marker), α 1,2-mannosidase (a Golgi marker), and GluR2. In the mitochondrial fraction (pellet 2), we detected calnexin, Grp75, PSD95, α 1,2-mannosidase, and GluR2. In the light membrane/cytosol fraction (supernatant 3), we detected Grp75, and EEA1 (an early endosome marker). The putative ER fraction (pellet 4) contained high levels of calnexin as well as GluR2, which is expected to be in the ER [23]–[25].

**Figure 3:**
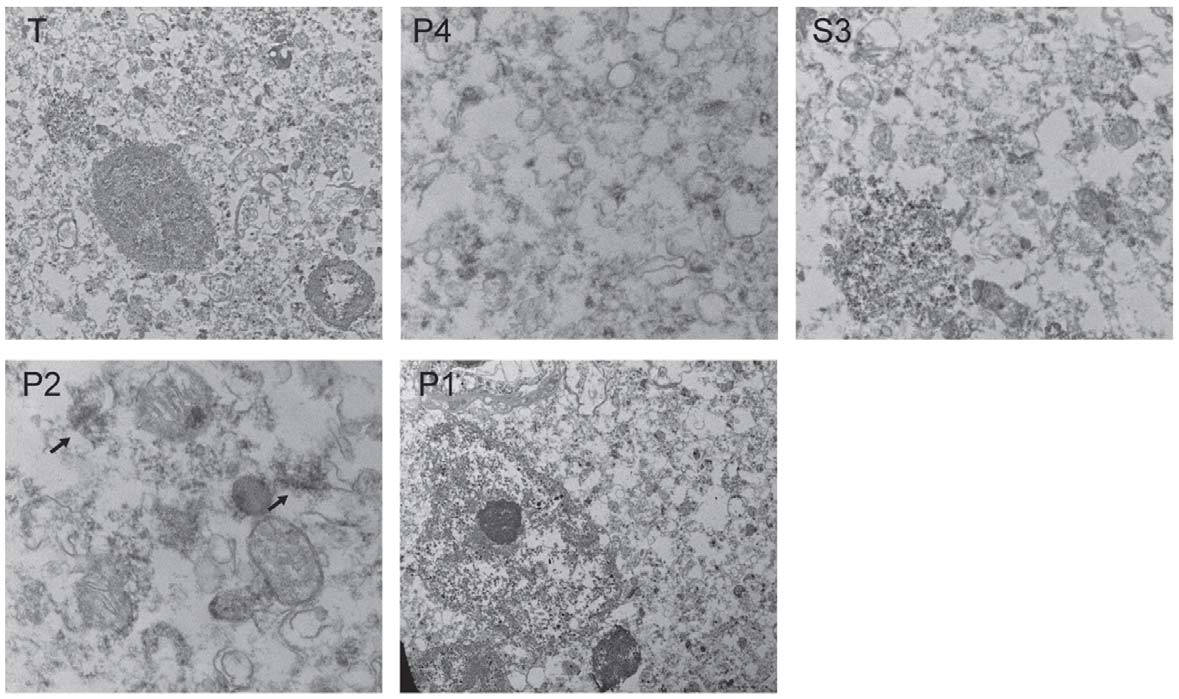
Assessment of enriched subcellular fractions isolated from postmortem human frontal cortex by electron microscopy. 100 mg of total unfractionated sample (T, 1650x magnification), endoplasmic reticulum (P4, 11,000x), light membranes and cytosol (S3, 4,400x), mitochondria (P2, 11,000x), and nuclei (P1, 1650x) were processed for electron microscopy. The ER and L/C fractions do not have intact Mit, Nuc, or postsynaptic density (PSD)-like structures. The Mit and Nuc fractions have PSD-like structures (black arrows).

Enriched subcellular fractions were further characterized using electron microscopy (Figure 3). The nuclear fraction (pellet 1) contained an enrichment of nuclei as well as postsynaptic density-like structures. The mitochondrial fraction (pellet 2) contained an enrichment of mitochondria and postsynaptic density-like structures. The light membrane/cytosol fraction (supernatant 3) was void of mitochondria, nuclei, or postsynaptic density-like structures. As expected, pellet 4 largely contained structures resembling ER microsomes and did not contain mitochondria, nuclei, or postsynaptic density-like structures.

### AMPA Subunit Protein Expression in Total Homogenate

We measured the expression by Western blot analysis of the AMPA receptor subunits, GluR 1–4, in total homogenate samples. Protein expression in the total homogenate was measured relative to the amount of protein loaded. We found no significant changes in GluR1, GluR2, GluR3, or GluR4 in the total homogenate in the schizophrenia group compared to the comparison group. We found no significant correlations between protein expression and age, pH, or PMI. In addition, we found no influence of sex on the expression of these subunits.

### AMPA Subunit Protein Expression in Enriched Endoplasmic Reticulum

**Figure 4:**
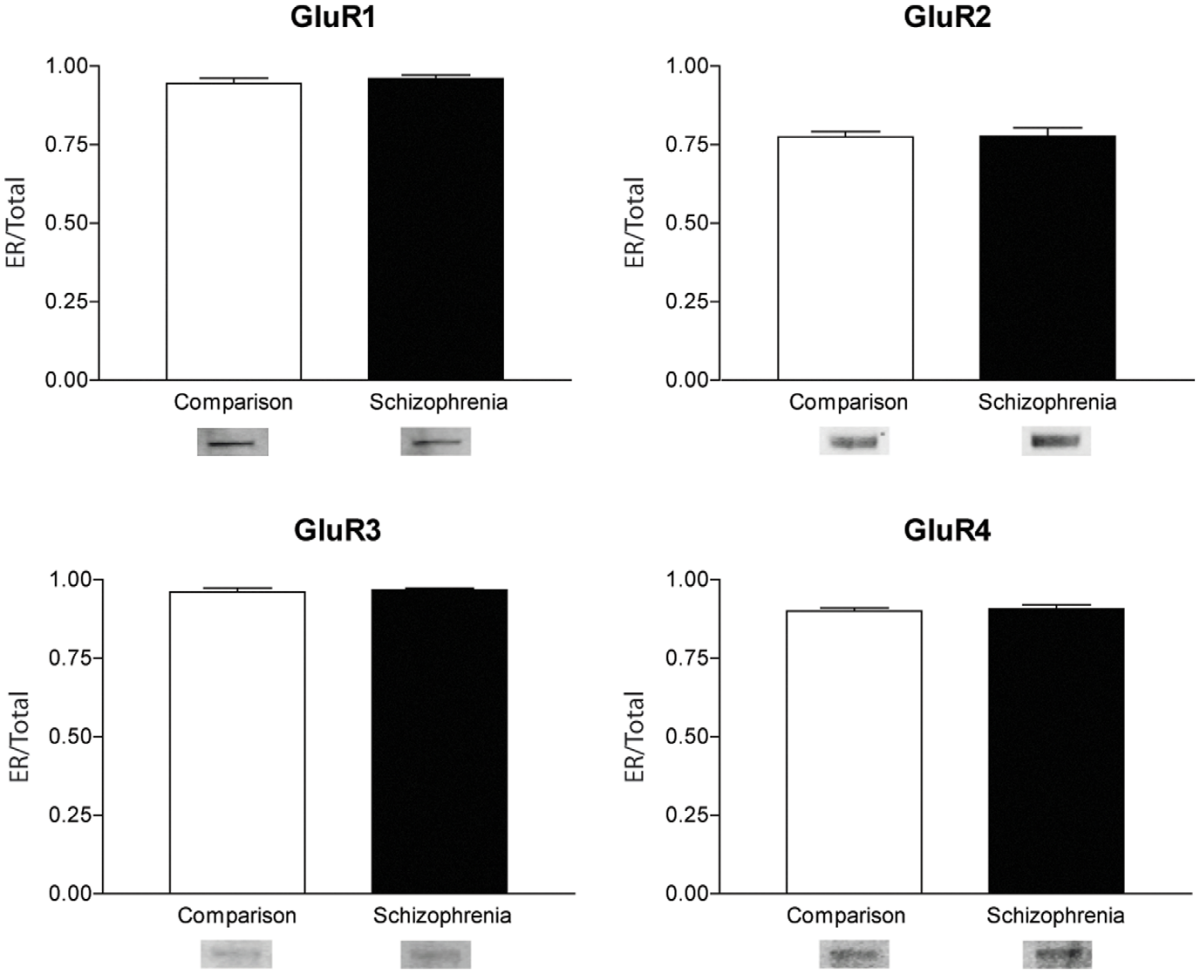
Western blot analysis of AMPA Receptor subunit expression in enriched endoplasmic reticulum fraction (A – GluR1, B – GluR2, C – GluR3, D – GluR4) normalized to relative expression in total homogenate. Representative blots of ER fraction. Error bars represent standard deviation. GluR1: F(1,30) = 0.57558, p = 0.45. GluR2: F(1,30) = 0.00725, p = 0.93. GluR3: F(1,30) = 0.28370, p = 0.59. GluR4: F(1,32) = 0.15205, p = 0.69.

We examined the expression of the AMPA receptor subunits, GluR 1–4, in the ER fraction (Figure 4). Protein expression in the enriched ER samples was measured relative to the corresponding protein in the total homogenate lane. We found no significant changes in GluR1, GluR2, GluR3, or GluR4 in the enriched ER fraction in the schizophrenia group compared to the comparison group. We found no significant correlations between protein expression and age, pH, or PMI in our enriched ER samples. In addition, we found no influence of sex on these protein levels.

In order to assess the validity of our normalization strategy, we also normalized expression of GluR3 and GluR4 to the intra-lane expression of the ER chaperone protein Jena Muenchen antibody 4 (JM4). The expression of JM4 was not changed in either the total homogenate or the ER fraction. We found no significant changes in GluR3 or GluR4 expression normalized to JM4 in the enriched ER fraction (data not shown). Due to technical limitation we were unable to perform these analyses for GluR1 and GluR2. We found no significant correlations between protein expression (normalized to JM4) and age, pH, or PMI and no influence of sex on protein levels in our enriched ER samples.

## Discussion

We have previously reported increased expression in DLPFC in schizophrenia of SAP97 and GRIP1, two proteins involved in the forward trafficking of AMPA receptors from the ER to the synapse [5]. We also found an increase in GluR1 protein expression in one distal compartment, in a cohort where we did not find changes in total GluR1 expression [5]. Taken together, these previous findings suggest alterations in the subcellular localization of AMPA receptors in schizophrenia. To extend this hypothesis, we measured the expression of the AMPA receptor subunits, GluR1–4, in an ER enriched fraction from human brain.

Based on Western blot protein characterization and electron microscopy, we have identified contents of the four fractions obtained using our differential sucrose gradient centrifugation protocol. In our ER fraction we found proteins predicted to be in the ER (calnexin and GluR2) but no markers from other compartments (histone3, GRP75, EEA1, PSD95, and α 1,2-mannosidase). The Western blot and electron microscopy profile of supernatant 3 implies that this fraction contains light membranes and cytosol. The presence in this fraction of Grp75, a stress protein typically found in the mitochondrial matrix, may indicate a loss of integrity of mitochondria either during the postmortem interval or processing of the samples. We found that pellet 2 contained post-synaptic densities and intact mitochondria while pellet 1 contained post-synaptic densities and intact nuclei. There is evidence of contamination in these fractions with calnexin and α 1,2-mannosidase, suggesting some breakdown of ER and mitochondrial membranes. Importantly, our fraction of interest, the ER fraction, is generally free from contamination by other subcellular organelles, including mitochondria, nuclei, and postsynaptic densities.

Compared to a previous report of ER isolation using immunoprecipitation with magnetic beads, the ER isolation protocol used in this study yields an ER fraction the has been verified to be free from contaminants by Western blot and electron microscopy [4]. While both studies provide the ability to measure the relative amount of glutamate receptor subunits within an ER fraction, the technique used in this study suggests that the sample is not contaminated by Golgi or other membranes [4].

Previously, we found an increase in GluR1 in early endosomes [5]. We also examined the expression of AMPA receptor subunits in late endosomes and found no change in expression [22]. Other studies using the same subject cohort found evidence of alterations in the proteins involved in the trafficking, expression, and signaling of the AMPA receptor subunits [7]. Thus, we expanded the examination of the subcellular localization of the AMPA receptors in schizophrenia. We found no change in the total GluR1–4 AMPA receptor subunit expression in schizophrenia, suggesting that the total expression of AMPA receptor subunits is unchanged between the groups. Using the expression of AMPA receptor subunits in total homogenate as a control, we measured the AMPA receptor subunits in the ER fraction relative to the amount in the homogenate. We found no change in the expression of GluR1–4 in the ER in schizophrenia, suggesting that although AMPA receptor interacting proteins responsible for the trafficking of AMPA receptors from the ER to the synapse are increased in schizophrenia, the expression of the AMPA receptor subunits themselves is not altered in the ER in this illness.

To assess the validity of our normalization strategy, we also analyzed the expression of GluR3 and GluR4 relative to the same-lane expression of the ER chaperone protein JM4 [26]. JM4 is a good candidate for normalization as it is ubiquitously expressed in ER and was unchanged in schizophrenia (data not shown) in both the ER fractions and the total homogenate samples. Similar to the results normalized to total GluR subunit expression, we found no significant change in the expression of GluR3 or GluR4 normalized to JM4. Since both normalization strategies yielded the same results for GluR3 and GluR4, we propose that our primary normalization strategy may be a valid approach for fractionation studies utilizing postmortem tissues.

Similar to other studies using postmortem tissue, there are technical limitations associated with this work. It is conceivable that small changes below our level of detection may be present in schizophrenia and our negative findings are the result of a Type II error. Further, while there was no correlation with expression of any of the subunits with age, all of the subjects included in this study were from an older population, and these findings may not be generalizable to younger patients. The effect of antipsychotic treatment was not assessed in this study because of the small number of available patients off antipsychotic medications at the time of death. It has been shown previously that prolonged treatment of rodents with antipsychotics can alter the expression of some AMPA receptor subunit mRNA and protein expression [27]–[29]. Thus, we cannot exclude that this lack of an effect might be due to normalization of these subunits by antipsychotic treatment.

In summary, we found no significant changes in the expression of AMPA receptor subunits in the ER in DLPFC in schizophrenia. In our previous studies, we examined distal AMPA receptor trafficking in schizophrenia, while in the current study we examined proximal localization of AMPA receptors. With the exception of our earlier GluR1 finding in early endosomes, our findings suggest that proximal and distal subcellular localization of AMPA receptors is largely unchanged in schizophrenia. However, AMPA receptor interacting proteins responsible for the trafficking of the receptors, SAP97 and GRIP1, are increased in schizophrenia. [5]. It may be that the function of these proteins relative to AMPA receptor trafficking is intact, but other modalities of trafficking involving these proteins are disturbed in this illness. Alternatively, the increase in SAP97 and GRIP1 may be counteracted by a previously reported decrease in PICK1, another AMPA receptor interacting protein involved in trafficking of the receptors [7]. In the present manuscript, we did not measure expression of AMPA interacting proteins localized to the ER, but it must be considered that changes in interacting protein expression may be altered in an enriched ER fraction. Further refinement of the isolation technique and secondary protein expression analysis may also allow for the measurement of post-translational modifications of AMPA receptors as well as determining interactions between AMPA receptors in the ER and as they are trafficked in the neurons.

Recent literature has suggested that the AMPA receptor subunits may be variably trafficked within the dendrites [30]. It has been suggested that the trafficking of AMPA receptors containing GluR1 subunits may follow the canonical trafficking modality while the receptors containing GluR2 subunits may be trafficked via dendrite-localized ER [31]. We hypothesized that trafficking of AMPA receptor subunits was altered in schizophrenia. The complex trafficking system suggesting alternative routes for individual AMPA receptor subunits further exemplifies the need to look at the contents of each subcellular compartment that is involved in AMPA receptor trafficking. Importantly, the subcellular expression of the AMPA receptors in isolated recycling endosomes and the postsynaptic density has not been examined in schizophrenia, areas that we are actively pursuing.

## Materials and Methods

### Subjects

Subjects from the Mount Sinai Medical Center brain bank were recruited prospectively and underwent extensive antemortem diagnostic and clinical assessment (Table 1). Exclusion criteria included a history of alcoholism, substance abuse, death by suicide, or coma for >6 h before death. Consent was obtained from next of kin for each subject. Brains were collected and cut coronally into 10 mm slabs. The left dorsolateral prefrontal cortex was dissected from the coronal slabs, snap frozen, and stored at −80°C. This tissue was pulverized, adding small amounts of liquid nitrogen as necessary, and stored at −80°C until used. Each of the eighteen (18) subjects with schizophrenia was matched for sex, and as closely as possible, for age and tissue pH with one comparison subject. Subjects groups did not significantly differ in mean age or tissue pH (Table 2). Subject groups did significantly differ in length of postmortem interval; however PMI was not correlated with expression of any of these subunits.

**Table 1:** Paired Subject Demographics.

Pair	Subject	Sex/Age, y	pH	PMI, h
1	Comparison	F/63	6.20	20.2
	Schizophrenia	F/62	6.74	23.7
2	Comparison	F/73	6.98	3
	Schizophrenia	F/71	6.60	5.5
3	Comparison	F/74	6.32	4.7
	Schizophrenia	F/75	6.49	21.5
4	Comparison	F/76	6.46	4.2
	Schizophrenia	F/77	6.01	9.7
5	Comparison	F/79	6.38	10.1
	Schizophrenia	F/79	6.80	9.9
6	Comparison	F/81	6.37	19.4
	Schizophrenia	F/81	6.67	15.1
7	Comparison	F/85	6.30	4.3
	Schizophrenia	F/84	6.64	NA
8	Comparison	F/96	6.30	4.5
	Schizophrenia	F/89	6.20	9.6
9	Comparison	M/59	6.67	20.4
	Schizophrenia	M/57	6.40	20.6
10	Comparison	M/65	6.82	3.8
	Schizophrenia	M/66	6.50	12.1
11	Comparison	M/69	6.67	7.4
	Schizophrenia	M/70	6.35	7.1
12	Comparison	M/73	6.17	14.9
	Schizophrenia	M/73	7.30	11.7
13	Comparison	M/76	6.32	2.9
	Schizophrenia	M/76	6.70	16.5
14	Comparison	M/84	6.83	11.4
	Schizophrenia	M/84	6.71	17.7
15	Comparison	M/93	6.28	4.1
	Schizophrenia	M/92	6.67	17.7
16	Comparison	M/68	6.55	2.7
	Schizophrenia	M/70	6.36	17.3
17	Comparison	M/75	6.43	5.0
	Schizophrenia	M/78	6.64	26.1
18	Comparison	M/95	6.53	4.1
	Schizophrenia	M/97	6.50	9.2

Abbreviations: Years (y), Postmortem Interval (PMI). Hours (h). Female (F). Male (M). Not available (NA).

**Table 2:** Pooled Subject Demographics.

Parameter	Comparison Subjects	Subjects with Schizophrenia
Sex, M/F	10/8	10/8
Age, years	76.8±10.6	76.7±10.3
pH	6.52±0.23	6.51±0.21
PMI, hours	8.2±6.3	14.8±6.8

Abbreviations: Male (M). Female (F). Postmortem Interval (PMI). Data are presented as mean ± SD.

### ER Isolation

Isolation of an ER enriched fraction was performed in parallel for each pair of subjects, one control and one schizophrenia subject, using nitrogen cavitation and differential sucrose gradient centrifugation (modified from [32]) (Figure 1). A starting amount of about 65 mg of pulverized tissue was used for each subject. All steps were carried out on ice or at 4°C for the centrifugation steps. Pulverized tissue was reconstituted in 1.2 mL of 1X ER extraction buffer (Sigma Aldrich) and subjected to nitrogen cavitation at 450 psi for 8 minutes. A 60 µL aliquot of this was saved as total homogenate. A low speed centrifugation (700×g) for 10 minutes was used to remove intact nuclei and large cellular debris (pellet 1). A subsequent 15000×g centrifugation for 10 minutes of the supernatant (supernatant 1) was done to pellet intact mitochondria (pellet 2). The resulting supernatant (supernatant 2) was loaded onto a three-layered sucrose gradient (2.0 M sucrose, 1.5 M sucrose, and 1.3 M sucrose) and centrifuged at 126000×g for 70 minutes on a Beckman L8-M ultracentrifuge in a SW60Ti rotor (Beckman). Following this centrifugation, the upper 200 µL of supernatant was collected (supernatant 3) and 100 to 300 µL of a dense white band between the top layer and the 1.3 M sucrose layer was collected (pellet 3). Pellet 3 was gently mixed by inversion with ice cold MTE buffer (premixed with 200 mM PMSF buffer). This mixture was centrifuged at 126000×g for 45 minutes resulting in a large, translucent pellet (pellet 4).

For each subject, pellets 1 and 2 were each reconstituted in 500 µL of PBS and pellet 4 was reconstituted in 50 µL of PBS. All samples were stored at −20°C until ready for use.

### Electron Microscopy

To visualize the content and confirm morphology of each fraction, samples were prepared for electron microscopy as previously described [5]. Briefly, fractions were fixed with 4% glutaraldehyde in 0.1 M cacodylate buffer (pH 7.4) overnight at room temperature. Samples were then washed and treated with 1% osmium tetroxide for 1 h, mordanted with 0.25% uranyl acetate in acetate buffer for 30 min to overnight, washed and dehydrated with a graded series of ethanol washes and propylene oxide. Finally, the samples were embedded in epoxy resin, thin sectioned and counterstained with uranyl acetate and lead citrate. Images were captured using an FEI Tecnai Spirit 20–120kv Transmission Electron Microscope.

### Western Blot Analysis

Prior to Western blot analysis, the amount of protein in each fraction was measured using a BCA protein assay kit (Thermo scientific). For each pair of subjects, samples for Western blot were prepared in duplicate with 5–10 µg of protein for each fraction placed in reducing buffer containing β-mercaptoethanol and heated at 70°C for 10 minutes. Protein loaded was consistent for each fraction within subject pairs, but not necessarily between subject pairs. Samples were then run by SDS-PAGE on Invitrogen (Carlsbad, California) 4–12% gradient gels, and transferred to PVDF membrane using Bio-Rad semi-dry transblotter (Hercules, California). Membranes were blocked in LiCor (Lincoln, Nebraska) blocking buffer for 1 h at room temperature before overnight incubation at 4°C with commercially available antibodies in 0.1% Tween LiCor blocking buffer with antisera dilutions determined empirically (Table 3). Following incubation, membranes were washed four times for 5 minutes each with PBS. Membranes were probed with IR-dye labeled secondary antibody in 0.1% Tween, 0.01% SDS LiCor blocking buffer for 1 h at room temperature in the dark. Membranes were washed again with PBS four times for 5 minutes each and then briefly rinsed three times in distilled water. The blots were stored in distilled water at 4°C until scanned using the LiCor Odyssey laser-based image detection method [33], [34].

**Table 3:** Antibodies used for western blots

Antibody	Species	Concentration	Company
Calnexin	Rabbit	1∶5000	CalBiochem, Gibbstown, NJ
Histone3	Rabbit	1∶1000	Cell Signaling, Danvers, MA
Grp75	Rabbit	1∶5000	Abcam Inc., Cambridge, MA
EEA1	Mouse	1∶500	BD Bioscience, San Jose, CA
PSD95	Mouse	1∶1000	Millipore, Bellarica, MA
α 1,2-Mannosidase	Rabbit	1∶1000	Abcam Inc., Cambridge, MA
GluR1	Rabbit	1∶200	US Biological, Swampscott, MA
GluR2	Mouse	1∶1000	US Biological, Swampscott, MA
GluR3	Rabbit	1∶200	US Biological, Swampscott, MA
GluR4	Goat	1∶100	Santa Cruz Biotechnology, Santa Cruz, CA

Abbreviations: Glucose Regulated 75 kDa Protein (GRP75). Early Endosome Antigen 1 (EEA1). Postsynaptic Density 95 (PSD95). Ionotropic Glutamate Receptor 1 (GluR1). Ionotropic Glutamate Receptor 2 (GluR2). Ionotropic Glutamate Receptor 3 (GluR3). Ionotropic Glutamate Receptor 4 (GluR4).

### Data Analysis

Image analysis from ER isolation Western blot studies was performed as previously described [5], [34]. Briefly, near-infrared fluorescent signals obtained from the LiCor Odyssey scanner were expressed as raw integrated intensity with top-bottom median intralane background subtraction using Odyssey 3.0 analytical software (LiCor). Protein band intensity for each protein in the ER isolation was normalized to the protein band intensity for the same protein in the total homogenate lane. All statistical analyses were performed using Statistica 7.1 (Statsoft, Tulsa, Oklahoma). Correlation analyses were carried out to identify any associations between the dependent variables and pH, age, and postmortem interval. One-way analysis of covariance (ANCOVA) was performed to analyze differences in GluR1–4 expression if significant correlations were found. If no correlations were present, data were analyzed with one-way analysis of variance (ANOVA). An additional secondary analysis was performed using sex as the independent measure.
